# Internal Standards for Quantitative Analysis of Chemical Warfare Agents by the GC/MS Method: Nerve Agents

**DOI:** 10.1155/2020/8857210

**Published:** 2020-08-11

**Authors:** Tomas Capoun, Jana Krykorkova

**Affiliations:** Ministry of Interior—Directorate General of the Fire Rescue Service CR, Population Protection Institute, Na Luzci 204, Lazne Bohdanec 533 41, Czech Republic

## Abstract

General conditions and requirements for an internal standard useful in the determination of chemical warfare agents (CWAs) by the method of gas chromatography coupled with mass detection (GC/MS) were defined. The determination is based on a GC/MS analysis of a mixture of a CWA with an internal standard, conversion of the TIC chromatogram to a chromatogram extracted at a particular *m*/*z* ratio, and calculation of the CWA concentration from the internal standard concentration, response factor, and chromatographic peak areas. Available internal standards were identified, and they were verified for seven organophosphorus nerve-paralysing agents. Corresponding response factors were determined as a ratio of gradients of the linear functions of the peak area and compound concentration. Linearity, repeatability, and accuracy of the measurements were evaluated. The determination can be performed on all GC/MS systems of the Fire Rescue Service of the Czech Republic (FRS), where no CWA standards are available.

## 1. Introduction

According to the Czech Republic law, competences of the FRS include chemical countermeasures in case of CWA spills or abuse. The countermeasures include chemical reconnaissance, detection, identification, and determination of CWAs. This activity is ensured by specialized FRS chemical laboratories. All laboratories are equipped by gas chromatography with mass detection (GC/MS) as most frequent analytical systems. Three types of systems are used in the FRS: GC/MS 7890A/5975 (Agilent), GC/MS Intuvo 9000/5977B of the same manufacturer, and EM 640 (Bruker Daltonik). Usually, quantitative analysis is performed by the absolute calibration method on these machines, but only for those analytes where a standard of corresponding purity is available.

The fundamental issue is that pure and certified CWA standards are not available in the Czech Republic. Therefore, we had to turn our attention to a procedure based on an internal standard, i.e., addition of a known amount of a substance different from the analyte into the sample, to determine these substances. This method is based on the fact that within a certain concentration range, the ratio of chromatographic peak areas and concentrations is constant. This ratio is called the response or calibration factor [1]:

(1) $$\begin{array}{c} F_{R}=\frac{A\;\mathrm{CWA}\;/c\text{ CWA}}{A\text{ ISTD}/c\text{ ISTD}\;}=\frac{A\text{ CWA}\cdot c\text{ ISTD}}{A\text{ ISTD}\cdot c\text{ CWA}}, \end{array}$$

where **F**_**R**_ is the response factor, **A**_CWA_ is the CWA chromatographic peak area, **A**_ISTD_ is internal standard chromatographic peak area, **c**_CWA_ is the CWA concentration in the solution, and **c**_ISTD_ is internal standard concentration in the solution.

The internal standard method has several significant advantages. Unlike the absolute calibration and standard addition methods, pure analyte standard is not required. The absolute calibration and standard addition methods include analysis of two separate samples which often introduces significant errors into the results [1, 2]. The same applies to the external standard method.

When using the internal standard method, the response factor value needs to be known in order to arrive at reliable analytical results. Additionally, the concentration range where the analyte and internal standard chromatographic peak area is a linear function must be known, as only then, the constant value of the response factor is ensured [1, 3]. This explains why many articles focusing on internal standard applications begin by a detailed validation, especially testing linearity of the function and the limit of quantification and repeatability of the analysis [4–16]. In case the sample is modified before the analysis (extraction, distillation, and solvent evaporation), maximum similarity of chemical and physical characteristics of the analyte and of the internal standard should be ensured [1, 3]. Close retention times of the analyte and the internal standard are also required in order to eliminate peak area discrimination at varying temperatures under temperature-programmed conditions [1, 3]. On top of these published requirements for the internal standard selection, one needs to consider the requirement that the internal standard must neither react or interact with the analyte nor constitute a decomposition product or other admixture of the analysed compounds.

For use in quantitative GC/MS analysis, the most efficient and reliable solution is the use of such internal standard which is identical or analogous to the analyte and labelled by a stable isotope [1, 17]. Examples include the determination of acrylamide in wheat samples, using [13C3]-acrylamide [18], determination of chrysene in a foil using [2H12]-chrysene [19], determination of rotundone in grapes and wine by the SPME method using [2H5]-rotundone [2], and determination of ethyl carbamate using [2H5]-ethyl carbamate, also in wine [9]. Additional examples include the use of [2H8]-dibenzothiophene for the determination of dibenzothiophene in crude oil, coal, and sediment extracts [20], [2H7]-meprobamate for the determination of meprobamate in blood [13], [13C12]-triclosane for the determination of triclosane in water [21], or [13C]-dichlorodiphenyltrichloroethane for the determination of dichlorodiphenyltrichloroethane (DDT) in the air [16]. However, internal standards labelled by a stable isotope are also used for samples where compounds other than analogues of the standard are analysed. For the determination of a number of hydrocarbons and other volatile compounds in internal or external air, [2H6]-benzene [11], [2H8]-toluene [22, 23] or [2H10]-ethylbenzene are used as internal standards. Similarly, some deuterized components of gasoline were used for the determination of aliphatic and aromatic hydrocarbons in water by the SPME method [7]. Additional examples of the internal standard use are [2H4]-1,2-dichloroethane for the determination of 26 halogenated compounds in water [24], [2H5]-3,4-methylenedioxyamphetamine and [2H6]-hydromorphone for the determination of narcotics and their metabolites in biological samples [4], and [2H14]-trifluarine, [2H6]-transpermethrine, and [2H4]-nitrophenol for the determination of 28 pesticides in the air [25]. This makes labelled analyte analogues ideal as internal standards for the determination of compounds by the GC/MS methods; however, their disadvantage is poor availability and high price.

When the internal standard differs from the analyte, compound losses during various phases of sample preparation unavoidably differ [1]. These differences can be efficiently minimized by the use of double internal standards, when two neighbouring representatives from the homologous series are used as internal standards for the determination of a specific agent [1, 19]. Further examples include determination of tocopherol in plasma, where pentamethylchromanol was used as the internal standard [6], determination of 2,5-di-tert-butyl-3-methylphenol in chewing gums using 3,5-di-tert-butylphenol [26], determination of furaneol in tomatoes using maltol [27], or determination of carisoprodole in blood using benzyl carbamate [13]. Anisole was used for the determination of various air contaminants [28]. In the analysis of food industry raw materials and products, 5-nonanone was used for the determination of 34 different chemical compounds in honey by the dynamic headspace method [5, 27], dinonyl phthalate for the determination of policosanol components extracted from rice bran wax [29], octyl acetate for the determination of 35 volatile compounds in essential oils obtained by steam distillation of lemon tree leaves and bark [30], and crotonic acid for the determination of volatile fatty acids in cheeses after steam distillation [31]. In water analysis, fluorobenzene was used as the internal standard for the determination of trihalomethanes by the GC/MS method using SPME [32] and benzyl benzoate for the determination of phthalates using the same method [33]. In the analysis of industrial products, diphenyl ether was used for the determination of lactide monomer in polylactic materials [34], 1,2-dichlorobenzene was used for the determination volatiles in water-oil emulsion by the SPME method [35], and tetrabromodiphenyl ether was used for the analysis of fire retarders on the basis of polybrominated diphenyl ethers, organophosphates, and brominated aromatic hydrocarbons [15].

This work focuses on the determination of CWAs by the GC/MS method. Only few sources discussing internal standards for this purpose could be identified. Dipinacolyl methyl phosphonate was described as an internal standard for the determination of tabun, cyclosarin, the VX agent, and nitrogen mustard [8]. The TNO laboratory in Rijswijk, the Netherlands, focusing especially on the CWA analysis, uses deuterized sulphur mustard, [2H8]-bis-(2-chloroethyl)sulphide, as internal standard for the determination of sulphur mustard [36].

When applying the internal standard, it is always necessary to evaluate the intensity of the analyte and standard chromatographic peaks. The TIC area is usually considered [1]. More precise results can be achieved when considering the peak area of a particular ion [2, 4], although the TIC area of the chromatographic peak can be sometimes more useful [5]. This is discussed in detail in publication [4].

This work focuses on a search for an available standard which would fulfill the requirement of matching CWA and internal standard response ratios on all GC/MS systems in the FRS laboratories. The aim of the work was to develop a procedure for the FRS laboratories which would not only allow for the determination of CWAs in solutions, but could be also used for a fast and simple determination of the active substance in the CWA preparates themselves. These preparates are further used for the calibration of the existing internal procedures for the determination of CWA, mostly by photometric and biochemical methods.

## 2. Materials and Methods

### 2.1. Chemicals

The quantitative analyses procedures by the GC/MS method were developed for the following CWAs: O-ethyl-N,N-dimethylphosphoramidocyanidate (tabun, GA, 88%), O-isopropylmethylphosphonofluoridate (sarin, GB, 64%), O-(3,3-dimethyl-2-butyl)methylphosphonofluoridate (soman, GD, 79%), O-cyclohexylmethylphosphonofluoridate (cyclosarin GF, 58%), O-ethyl-S-(diisopropylaminoethyl)methylphosphonothioate (VX agent, 69%), O-ethyl-S-(diethylaminoethyl)methylphosphonothioate (Edemo, 51%), and O-ethyl-S-(dimethylaminoethyl)methylphosphonothioate (Medemo, 24%). All compounds were prepared in VOZ Zemianské Kostoľany, Slovakia. Tabun assay was determined by potentiometric argentometric titration of cyanides by silver nitrate indicated by a silver electrode; assay of sarin, soman, and cyclosarin was determined by potentiometric lanthanometric titration of fluorides by lanthanum (III) chloride indicated by a fluoride ion selective electrode; assay of the VX agent, Edemo, and Medemo compounds was determined by potentiometric argentometric titration of thiols by silver nitrate indicated by a sulphide ion selective electrode.

Triethylphosphate (99.8+%, Sigma-Aldrich), heptan-1-ol (>99%, Fluka), tri-n-butylphosphate (p.a., Merck), di-n-hexylamine (p.a., Merck), and di-n-amylether (p.a., Merck) were the internal standards used. Solutions of CWAs and internal standards were prepared in acetone or n-hexane (SupraSolv, for GC, Merck).

### 2.2. Measurement Conditions and Parameters

The measurements were performed on the following systems at conditions and parameters listed in Table 1. System A—GC/MS 7890A/5975C (Agilent Technologies, Inc., Wilmington, USA); system B—GC/MS Intuvo 9000/5977B same manufacturer; and system C—mobile GC/MS EM 640 (Bruker Daltonik GmbH, Bremen, Germany).

Solutions of CWAs and internal standard were mixed in a 1 : 1 (*v*/*v*) ratio, and the mixture was introduced into the injection inlet of the GC/MS system. The linearity range of the chromatographic peak area as a function of the compound concentration was studied in parallel both for the CWA and the corresponding internal standard. Hence, mixtures of CWAs and standards of the same concentration were injected. Triplicate measurements were performed for each concentration of the compound and the standard.

### 2.3. Chromatogram Evaluation

Peaks corresponding to the CWA and the internal standard were identified in the TIC chromatogram recorded in the scan mode. The peak area was obtained by integration using the evaluation software listed in Table 1. Generally, automatic integration was used, and only tailing peaks were integrated manually. For further study, EIC chromatograms at a particular *m*/*z* ratio were extracted from the TIC chromatograms; peak areas corresponding to the CWAs and the internal standards were obtained by an identical procedure.

In order to assess the dependency of the chromatographic peak area on the concentration of the given compound in the solution, calibration curves were constructed. The linearity range was determined using statistical software [37] based on the correlation coefficient *R* and coefficient QC values. Coefficient values of *R*_CRIT_ 0.99 and QC_CRIT_ 5.00 were considered as critical for the testing. The gradient, *y*-range, and standard deviation of the gradient and range were evaluated by software [37] in the identified linearity range.

In order to assess the accuracy of CWA determination, a series of results from parallel determinations was compared to the known concentration. The *t*-test was used for statistical evaluation [37], comparing the value of *t* criteria to the critical value. Based on the results from the parallel measurement, the precision of the determination was tested. The method of concentration levels from parallel measurements and calculation of relative standard deviation was selected for the statistical evaluation [37].

## 3. Results and Discussion

### 3.1. Study of Chromatographic Peak Area Dependence on Concentration

The primary aim of this work was to find a suitable internal standard, applicable on all GC/MS systems across the FRS chemical laboratories in a universal procedure. The procedure would be used especially for a quick and simple determination of the active ingredient of own CWA preparates which are then used for the calibration of the existing determination procedures. This means a binary mixture of the CWA and the internal standard is analysed, and hence, neither similarity of chemical properties of the analyte and the internal standard nor close physical characteristics are an issue here as these matter mostly in case of sample preparation before the analysis itself. On the other hand, this requires highly reliable determination which is closely related to the linearity of the chromatographic peak area as a function of the compound concentration.

Assuming a linear function of the chromatographic peak area and concentration, the relation can be described by the following equation:

(2) $$\begin{array}{c} A=k\cdot c+q, \end{array}$$

where **A** is the chromatographic peak area, **k** is the gradient, **c** is concentration, and **q** is the intercept on the peak area axis. This equation can be combined with the response factor equation (1):

(3) $$\begin{array}{c} F_{R}=\frac{\left(k\;\mathrm{CWA}\;\cdot c\text{ CWA}+q\text{ CWA}\right)\cdot c\text{ ISTD}}{\left(k\text{ ISTD}\cdot c\text{ ISTD}+q\text{ ISTD}\right)\cdot c\text{ CWA}}. \end{array}$$

Assuming that the intercept on the peak area is negligible compared to the product of gradient and concentration, i.e., the linear function of CWA and internal standard peak area and concentration passes through the origin, the response factor equals the ratio of gradients of the two linear functions of CWA and the internal standard peak area and concentration:

(4) $$\begin{array}{c} F_{R}=\frac{k\text{ CWA}}{k\text{ ISTD}}. \end{array}$$

**Figure 1:**
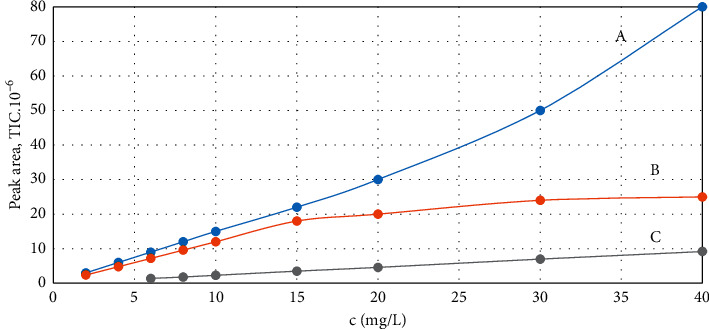
TIC chromatographic peak area of cyclosarin as a function of its concentration in the solution, measured at the following GC/MS systems: 7890A/5975C (A), intuvo 9000/5977B (B), and EM 640 (C).

Use of equation (4) for the determination of the response factor has two fundamental prerequisites. First, the intercepts on the peak area axis must be negligible compared to the product of the gradient and lower limit of the linearity range both for the CWA and for the internal standard. We have set the intercept must be lower than 10% of the product of gradient and the lowest useful concentration. Second, the response factor can only be applied in the concentration range where the function of CWA and the internal standard peak area is linear. The determination method was developed for the different GC/MS systems whose linearity ranges significantly differ. Moreover, the linearity range also depends on the analyte. Yet, linearity of the response greatly influences reliability of the determination. A typical example is illustrated in Figure 1 showing the chromatographic peak area of cyclosarin as a function of its concentration in the solution.

A number of potential compounds were tested during the search for suitable internal standards. Both TIC chromatograms and EIC chromatograms extracted for a characteristic ion *m*/*z* were evaluated. We have found that the calculation of the response factor from the TIC chromatograms recorded in scan mode is not suitable. Only few standards with an identical response factor across the tested systems could be found; most standards exhibited high differences between the systems. Moreover, a reproducible linear calibration curve could not be constructed at all for some of the compounds even in a narrow concentration range. In some cases, the requirement of a negligible intercept on the peak area axis versus the product of gradient and concentration was not met.

The use of EIC chromatogram, extracted both for the CWA and the internal standard at a particular *m*/*z* value, is the optimal procedure for CWAs determination using the internal standard method. This implies that an internal standard exhibiting an intense peak corresponding to the same ion as for the CWA mass spectrum has to be identified. In turn, this allows for a wider linearity range with the EIC chromatograms than with the TIC chromatograms. Moreover, the EIC chromatograms of the CWAs and the identified standards meet the negligible intercept requirement more easily. Reproducibility of the peak area readings is much higher for the EIC chromatograms than for the TIC chromatograms. Moreover, the EIC chromatogram peak area reading is more robust and resistant to interference with compounds with similar retention times than with the use of TIC chromatograms.

### 3.2. Determination of Tabun

The *m*/*z* values of 43, 70, 44, 133, and 162 dominate the mass spectrum. Methyllaurate (*m*/*z* 43) and crotonaldehyde (*m*/*z* 70) were tested as the available chemicals for the internal standard. However, the response factors highly varied across the GC/MS systems (the **F**_**R**_ values of methyllaurate *m*/*z* 43 reached 0.75, 0.27, and 1.02 for systems *A*, *B*, and *C*, respectively; for crotonaldehyde *m*/*z* 70, the values reached 0.64, 0.49, and 1.14 for systems *A*, *B*, and *C*, respectively). Although satisfactory results could be obtained when using dihexylamine at *m*/*z* 43 as the internal standard, we found out that as a weak base, dihexylamine causes the decomposition of tabun after a certain period of time, giving rise to O-ethyl-O-methyl N,N-dimethylamidophosphate. Hence, dihexylamine does not fulfill the requirement that the internal standard must not react with the analyte. Best results were achieved with heptan-1-ol at *m*/*z* 70, as shown in Table 2. For simplicity, chromatographic peak area values are given in millions of abundance units in all following tables.

### 3.3. Determination of Sarin, Soman, and Cyclosarin

**Figure 2:**
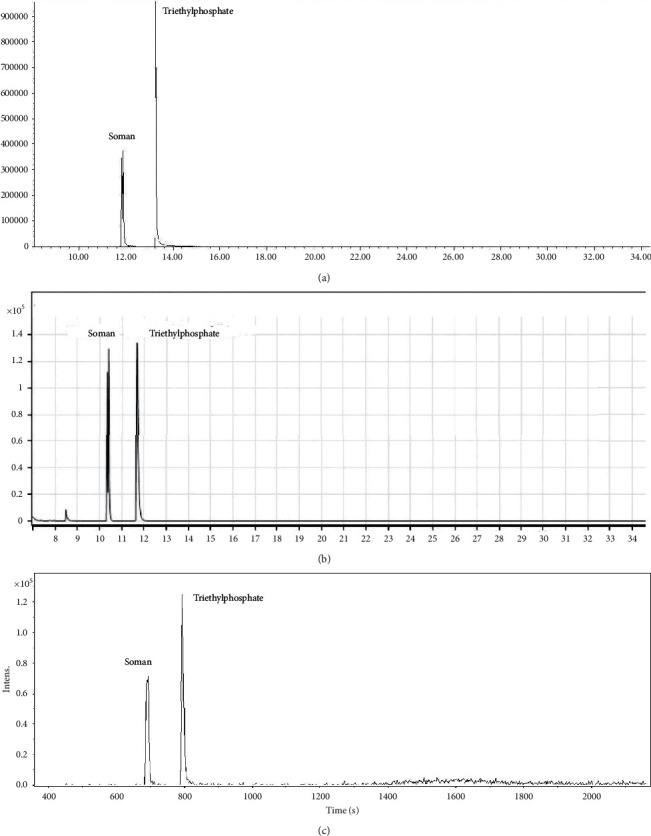
Examples of soman and triethylphosphate chromatograms extracted at *m*/*z* 99 measured on the following GC/MS systems: (a) 7890A/5975C, **c**_CWA_ = 10.7 mg/L, **c**_ISTD_ = 15.3 mg/L, (b) intuvo 9000/5977B, **c**_CWA_ = 7.3 mg/L, **c**_ISTD_ = 4.3 mg/L, and (c) EM 640, **c**_CWA_ = 39.0 mg/L, **c**_ISTD_ = 31.9 mg/L.

Other type G-type nerve-paralysing agents exhibit the *m*/*z* 99 as one of the most prominent mass spectrum peaks. This fact has led us to the selection of triethylphosphate as a suitable internal standard. The calculated response factor was identical across the three systems tested, as shown in the evaluation in Tables 3–5. The molecule of soman (Table 4) contains two asymmetric atoms, namely, phosphorus and carbon in the pinacolyl group, and forms four stereoisomers [38] giving rise to two chromatographic peaks. Examples are shown in Figure 2. The total area is a sum of both peak areas.

### 3.4. Determination of the VX Agent

The VX agent exhibits a dominant peak at *m*/*z* ratio 114 and less pronounced peaks at *m*/*z* 72 and 127. Tripropylamine (*m*/*z* 114 and 72) and 3-aminohexane (*m*/*z* 72) have much shorter retention time at the separation conditions used, compared to the VX agent. Further internal standards tested exhibited significant variances in the agent VX vs. internal standard response across the systems used. We have verified, e.g., m-chloroaniline (**F**_**R**_ value for *m*/*z* 127 reached 0.12, 0.03, and 0.65 for systems *A*, *B*, and *C*, respectively), 5-chloro-2-methoxypyrimidine (**F**_**R**_ value for *m*/*z* 114 reached 1.60, 0.30, and 1.21 for systems *A*, *B*, and *C*, respectively), 2-chloro-4-methoxypyrimidine (**F**_**R**_ value for *m*/*z* 114 reached 1.80, 0.50, and 1.42 for systems *A*, *B*, and *C*, respectively), and dimethyl adipate (**F**_**R**_ value for *m*/*z* 114 reached 3.00, 1.66, and 2.07 for systems *A*, *B*, and *C*, respectively). Very close response factor values across different GC/MS systems could be achieved with dipropyltryptamine at *m*/*z* 114 (**F**_**R**_ value reached 0.71, 0.72, and 0.73 for systems *A*, *B*, and *C*, respectively). However, taking into account its problematic nature, dipropyltryptamine was not selected as the internal standard for the VX agent.

Most reliable results could be achieved with di-n-hexylamine at *m*/*z* 114 as the internal standard. The evaluation is shown in Table 6. Analysis of the mixture of the VX agent and the internal standard must be performed in an apolar solvent. We found out that, in the methanolic environment, agent VX undergoes decomposition due to the presence of the basic amine, and the requirement that the internal standard must neither react with the analyte nor interact in any way is, therefore, not fulfilled. The whole amount of the VX agent was completely decomposed already 6 hours after mixing the methanolic solution of the VX agent and di-n-hexylamine in a concentration range 5–50 mg/L. For the analysis in methanolic solution, this implies that the analysed mixture would have to be mixed with the di-n-hexylamine solution immediately before the analysis, which is not advantageous for larger sample sets.

### 3.5. Determination of Edemo

The Edemo agent exhibits two dominant peaks corresponding to *m*/*z* ratios 86 and 99. The available compounds tested as internal standards are significantly more volatile than the analyte, and they have significantly shorter retention times: triethylamine (*m*/*z* 86), tripropylamine (*m*/*z* 86), 2-(diethylamino)ethan-1-ol (*m*/*z* 86), and triethylphosphate (*m*/*z* 99). Attention was also paid to N-butylacetamide at *m*/*z* 86; however, although reproducible results with close response factor values across the GC/MS systems could be obtained (**F**_**R**_ value reached 0.97, 0.93, and 0.94 for systems *A*, *B*, and *C*, respectively), the compound was not selected as the internal standard for Edemo determination due to its high price. Most reliable results were achieved with tri-n-butylphosphate as the internal standard at *m*/*z* 99 (Table 7).

### 3.6. Determination of Medemo

As regards to Medemo, as a dimethyl derivative of O-ethyl-S-(dialkylaminoethyl)methylphosphonothioate, the *m*/*z* values 58 and 71 dominate its mass spectrum. Tri-n-octylamine (*m*/*z* 71) was tested as an available compound. However, the linearity range of the chromatographic peak area as a function of concentration was very narrow, rendering the compound practically useless. Response factors of several other compounds tested significantly varied across the GC/MS systems used. We tested, for example, 3-aminohexane (**F**_**R**_ value for *m*/*z* 58 reached 0.69, 0.34, and 0.93 for systems *A*, *B*, and *C*, respectively) or ethylbutyrate (**F**_**R**_ value for *m*/*z* 71 reached 0.48, 0.18, and 0.40 for systems *A*, *B*, and *C*, respectively). Satisfactory results were achieved with di-n-amylether at *m*/*z* 71 as the internal standard, as shown in Table 8.

### 3.7. Validation of the Determination

Accuracy and precision of the determination were assessed by method validation. Accuracy was assessed by the statistical *t*-test. Solutions to be analysed were prepared from different CWA batches than those used for the determination of response factors. Five parallel determinations were performed for each solution. The obtained set of concentration determinations was, then, evaluated by statistical software [37] to assess the *t* criterion which was compared to the critical value. The procedure gives accurate results for *t* < *t*_CRIT_.

Precision was statistically assessed by the method of concentration levels from parallel measurements and calculation of relative standard deviations [37]. Precision was evaluated for the abovementioned sets of five results.

The accuracy and precision assessment are summarized in Table 9, indicating that the method yields accurate results and that the relative standard deviation does not exceed 15% which generally corresponds to the precision of GC/MC-based determination procedures. The highest values of relative repeatability were achieved on the EM 640 system. On the 7890A/5975C and Intuvo 9000/5977B GC/MS systems, the maximum relative precision of CWAs determination reached 7%.

### 3.8. Multilaboratory Comparison

The studied determination method could also be verified in a multilaboratory comparison performed as part of the FRS chemical laboratories proficiency testing. The investigation was performed in 2017–2019 with six chemical laboratories equipped with three 7890A/5975C GC/MS systems, five Intuvo 9000/5977B systems, and two mobile EM 640 systems. Accuracy of the results was evaluated by statistical software [37]. The value of the *z*-score as a ratio of the difference of known and determined concentration and standard deviation was calculated. Results with an absolute value of *z*-score below or equal to 2.0 were considered as accurate. A relative standard deviation of 12.5% was chosen for the calculation of the *z* value.

Evaluation of the multilaboratory comparison is summarized in Table 10. In total, 3.1% of inaccurate results were obtained during the analysis of five CWAs. The relative difference of known and determined concentration between the laboratories did not exceed 6%. Taking into account that participants of the multilaboratory comparison did not have standards of the nerve-paralysing agents available, these results can be considered very good.

## 4. Conclusions

A procedure based on the use of an internal standard in GC/MS analysis was developed to determine the concentration of nerve agents. We found that determination of response factors from TIC chromatograms recorded in the scan mode is not suitable. A standard which would have an identical response factor across the systems tested could only be identified in exceptional cases; in fact, the response factor values highly differed in most cases. An optimal procedure was to use the EIC chromatogram extracted for a particular ion, extracting both CWA and internal standard peaks at the same m/*z* ratio. Unlike TIC chromatograms, the EIC chromatograms exhibit linearity of the chromatographic peak area function of concentration in a much wider range, and the peak area readings are significantly more reproducible and robust against interference with compounds with close retention times.

We were able to identify and verify a standard which exhibits a pronounced peak corresponding to the same ion present in the CWA mass spectrum, and verified the useful concentration range for all nerve agents in scope of this work. The response factors for individual CWAs and internal standards were calculated as ratios of the gradient of linear functions of the peak area and concentration. The response factors are valid across all GC/MS systems within the FRS laboratories. A simple procedure for the determination, comprising the preparation of CWA and internal standard solutions, their mixing, and injection into the GC/MS system, was developed. The analysis is followed by identification of the components as usual, extraction of the chromatogram at a particular *m*/*z* value, integration, and reading of the chromatographic peak area of the analyte and the internal standard. The CWA concentration is, then, calculated based on the internal standard concentration, peak areas, and response factor.

The determination procedure was validated by accuracy and repeatability testing and by multilaboratory comparison. The validation showed that the procedure yields accurate results, and the relative standard deviation does not exceed 15% which corresponds to the precision of GC/MS-based determinations. During the multilaboratory comparison, only 3 out of 98 results of the five CWAs analysed were found inaccurate. The relative difference of known concentrations and concentration determined in the different participating laboratories did not exceed 6%.

The most important and decisive asset of this work is that the developed procedure enables the chemical laboratories to determine the concentration of CWAs in a solution in the absence of standards. This is especially important for the determination of purity of own CWA preparates which are, then, in turn used for the preparation of calibration solutions for other methods employed.

## Figures and Tables

**Table 1:** Measurement conditions and parameters.

GC/MS		7890A/5975C	Intuvo 9000/5977B	EM 640
Column		HP-5MS 30 m × 0.25 mm, 0.25 *μ*m	HP-5MS 30 m × 0.25 mm, 0.25 *μ*m	HP-5MS 25 m × 0.35 mm, 1 *μ*m
Carrier gas		Helium, 147 kPa constant pressure	Helium, 1.2 mL/min constant flow	Nitrogen, 500 hPa constant pressure
Sampler		Agilent GC 80	Agilent 7693A	—
Injection volume		1 *μ*l	1 *μ*l	1 *μ*l
Inlet		290°C, splitless mode, purge flow 100 mL/min at 2 min	290°C, splitless mode, purge flow 100 mL/min at 2 min	230°C, splitless mode, purge flow 30 mL/min at 1 min
Oven		40°C (2 min), 10°C/min to 280°C (10 min)	40°C (2 min), 10°C/min to 280°C (10 min)	40°C (2 min), 10°C/min to 280°C (10 min)
Detector		Quadrupole MS, EI, scan mode, transfer line 290°C, scan range 35–600 amu, solvent delay 6 min	Quadrupole MS, EI, scan mode, transfer line 290°C, scan range 35–600 amu, solvent delay 6 min	Quadrupole MS, EI, scan mode, transfer line 280°C, scan range 50–550 amu, solvent delay 6 min
Evaluation of chromatograms		Agilent chemstation GC/MSD—data analysis, version E.02.02., Agilent Technologies, Inc., 2011	MassHunter Workstation software, version B.07.00, Agilent Technologies, Inc., 2014	Bruker Data Analysis, version 1.1., Bruker Daltonik GmbH, 2003

**Table 2:** Evaluation of the chromatographic peak area of tabun and heptan-1-ol at *m*/*z* 70 as a linear function of their concentration (critical values of the correlation coefficient **R**_CRIT_ 0.99 and QC coefficient QC_CRIT_ 5.00).

GC/MS compound	7890A/5975C		Intuvo 9000/5977B		EM 640	
	Tabun	Heptanol	Tabun	Heptanol	Tabun	Heptanol
Retention time (min)	13.3	10.4	10.3	7.8	12.6	9.8
Linearity range (mg/L)	2.5–40	2.5–40	0.5–10	0.5–10	10–50	10–50
**R**	0.9988	0.9994	0.9999	0.9968	0.9999	0.9996
QC	3.28	2.56	0.86	4.13	0.21	1.49
Gradient (*A* × 10^−6^ × L/mg)	0.71	1.4	0.13	0.25	0.015	0.032
Intercept (*A* × 10^−6^)	0.065	−0.34	−0.0030	−0.0090	0.00012	−0.015
Response factor **F**_**R**_	0.49		0.52		0.48	
Average value of **F**_**R**_	0.50					

**Table 3:** Evaluation of the chromatographic peak area of sarin and triethylphosphate at *m*/*z* 99 as a linear function of their concentration (critical values of the correlation coefficient **R**_CRIT_ 0.99 and QC coefficient QC_CRIT_ 5.00).

GC/MS compound	7890A/5975C		Intuvo 9000/5977B		EM 640	
	Sarin	Triethyl-phosphate	Sarin	Triethyl-phosphate	Sarin	Triethyl-phosphate
Retention time (min)	7.4	13.2	5.1	11.7	7.1	13.3
Linearity range (mg/L)	1–40	2.5–40	0.5–10	0.5–15	5–40	5–50
**R**	0.9999	0.9986	0.9998	0.9996	0.9979	0.9988
QC	1.07	3.68	1.74	2.22	3.84	3.71
Gradient (*A* × 10^−6^ × L/mg)	2.9	1.4	0.74	0.38	0.040	0.020
Intercept (*A* × 10^−6^)	0.18	−0.24	−0.030	0.014	0.0088	0.0073
Response factor **F**_**R**_	2.06		1.93		1.97	
Average value of **F**_**R**_			1.99					

**Table 4:** Evaluation of the chromatographic peak area of soman and triethylphosphate at *m*/*z* 99 as a linear function of their concentration (critical values of the correlation coefficient **R**_CRIT_ 0.99 and QC coefficient QC_CRIT_ 5.00).

GC/MS compound	7890A/5975C		Intuvo 9000/5977B		EM 640	
	Soman	Triethyl-phosphate	Soman	Triethyl-phosphate	Soman	Triethyl-phosphate
Retention time (min)	11.7	13.2	10.3	11.7	11.6	13.3
Linearity range (mg/L)	2.5–20	2.5–40	1–15	0.5–15	5–40	5–50
**R**	0.9971	0.9985	0.9991	0.9989	0.9988	0.9978
QC	4.32	3.63	3.07	3.76	3.16	4.79
Gradient (*A* × 10^−6^ × L/mg)	1.6	1.4	0.45	0.39	0.024	0.021
Intercept (*A* × 10^−6^)	−0.65	−0.34	−0.030	−0.019	−0.0019	0.010
Response factor **F**_**R**_	1.10		1.16		1.16	
Average value of **F**_**R**_	1.14			

**Table 5:** Evaluation of the chromatographic peak area of cyclosarin and triethylphosphate at *m*/*z* 99 as a linear function of their concentration (critical values of the correlation coefficient **R**_CRIT_ 0.99 and QC coefficient QC_CRIT_ 5.00).

GC/MS compound	7890A/5975C		Intuvo 9000/5977B		EM 640	
	Cyclo-sarin	Triethyl-phosphate	Cyclo-sarin	Triethyl-phosphate	Cyclo-sarin	Triethyl-phosphate
Retention time (min)	14.6	13.2	12.5	11.7	14.7	13.3
Linearity range (mg/L)	5–30	2.5–40	1–12.5	0.5–15	5–50	5–50
**R**	0.9998	0.9992	0.9996	0.9998	0.9969	0.9996
QC	1.62	2.82	2.01	1.28	4.45	1.68
Gradient (*A* × 10^−6^ × L/mg)	3.2	1.5	0.78	0.38	0.046	0.021
Intercept (*A* × 10^−6^)	−1.3	−0.29	0.0061	−0.017	0.022	0.0077
Response factor **F**_**R**_	2.17		2.07		2.18	
Average value of **F**_**R**_			2.14			

**Table 6:** Evaluation of the chromatographic peak area of the VX agent and di-n-hexylamine at *m*/*z* 114 as a linear function of their concentration (critical values of the correlation coefficient **R**_CRIT_ 0.99 and QC coefficient QC_CRIT_ 5.00).

GC/MS compound	7890A/5975C		Intuvo 9000/5977B		EM 640	
	VX	Dihexyl-amine	VX	Dihexyl-amine	VX	Dihexyl-amine
Retention time (min)	22.1	17.0	18.6	14.4	23.8	17.7
Linearity range (mg/L)	7.5–20	7.5–20	7.5–20	7.5–20	15–50	15–50
**R**	0.9934	0.9981	0.9987	0.9938	0.9999	0.9999
QC	4.38	4.81	4.68	3.89	0.43	0.23
Gradient (*A* × 10^−6^ × L/mg)	0.38	0.67	0.24	0.45	0.020	0.036
Intercept (*A* × 10^−6^)	−0.21	−0.49	−0.031	0.033	0.0013	−0.0023
Response factor **F**_**R**_	0.57		0.54		0.55	
Average value of **F**_**R**_			0.55					

**Table 7:** Evaluation of the chromatographic peak area of edemo and tri-n-butylphosphate at *m*/*z* 99 as a linear function of their concentration (critical values of the correlation coefficient **R**_CRIT_ 0.99 and QC coefficient QC_CRIT_ 5.00).

GC/MS compound	7890A/5975C		Intuvo 9000/5977B		EM 640	
	Edemo	Tributyl-phosphate	Edemo	Tributyl-phosphate	Edemo	Tributyl-phosphate
Retention time (min)	20.5	21.2	16.4	17.1	18.9	19.6
Linearity range (mg/L)	5–30	5–30	2.5–15	2.5–12.5	10–50	10–50
**R**	0.9964	0.9996	0.9980	0.9967	0.9978	0.9981
QC	4.00	2.14	4.62	4.53	3.29	3.17
Gradient (*A* × 10^−6^ × L/mg)	1.1	4.2	0.16	0.61	0.014	0.054
Intercept (*A* × 10^−6^)	−0.18	−2.1	−0.012	−0.11	0.0017	0.0045
Response factor **F**_**R**_	0.25		0.27		0.26	
Average value of **F**_**R**_			0.26					

**Table 8:** Evaluation of the chromatographic peak area of Medemo and di-n-amylether at *m*/*z* 71 as a linear function of their concentration (critical values of the correlation coefficient **R**_CRIT_ 0.99 and QC coefficient QC_CRIT_ 5.00).

GC/MS compound	7890A/5975C		Intuvo 9000/5977B		EM 640	
	Medemo	Diamyl-ether	Medemo	Diamyl-ether	Medemo	Diamyl-ether
Retention time (min)	18.4	12.4	14.6	9.5	19.2	13.2
Linearity range (mg/L)	10–40	5–40	2.5–12	2.5–12	15–50	10–50
**R**	0.9988	0.9997	0.9997	0.9968	0.9995	0.9998
QC	2.58	1.77	0.81	4.01	1.68	0.79
Gradient (*A* × 10^−6^ × L/mg)	0.53	2.5	0.11	0.49	0.017	0.084
Intercept (*A* × 10^−6^)	−0.10	−1.3	−0.014	−0.0034	−0.0089	−0.00095
Response factor **F**_**R**_	0.21		0.22		0.20	
Average value of **F**_**R**_			0.21					

**Table 9:** Assessment of CWA determination accuracy and precision testing (**c**_ISTD_—internal standard concentration, *t*—*t* criterion value, *S*_*R*_—relative standard deviation, number of measurements *n* = 5, and *t*_CRIT_ = 2.776).

CWA/Internal standard	**c**_ISTD_ (mg/L)	Known CWA concentration (mg/L)	GC/MS system	Determined concentration (mg/L)	*t*	*S*_*R*_ (%)
Tabun/heptan-1-ol	30.0	32.7	7890A/5975C	29.9	2.147	4.0
			EM 640	31.6	1.152	11.1
	4.0	3.4	Intuvo 9000/5977B	3.6	1.263	2.2

Sarin/triethyl-phosphate	20.0	14.5	7890A/5975C	14.5	0.049	3.8
			EM 640	13.3	1.792	14.7
	5.0	9.1	Intuvo 9000/5977B	9.7	2.046	6.0

Soman/triethyl-phosphate	20.0	19.5	7890A/5975C	18.3	0.311	5.6
			EM 640	19.0	0.745	4.2
	12.0	7.0	7890A/5975C	7.5	1.538	5.4
			Intuvo 9000/5977B	6.7	1.147	3.7
			EM 640	7.1	0.297	9.3

Cyclosarin/triethyl-phosphate	25.0	21.0	7890A/5975C	20.3	0.459	5.6
			EM 640	20.7	0.612	4.2
	12.0	8.8	7890A/5975C	9.5	2.005	5.4
			Intuvo 9000/5977B	7.9	1.916	3.7
			EM 640	8.9	0.309	9.3
	5.0	3.7	Intuvo 9000/5977B	4.0	1.714	6.0

VX agent/di-n-hexylamine	15.0	17.7	7890A/5975C	17.0	0.699	5.6
			Intuvo 9000/5977B	16.7	1.015	4.9
			EM 640	15.3	1.973	8.4
	10.0	19.3	7890A/5975C	17.6	1.514	6.8
			Intuvo 9000/5977B	18.0	1.865	9.0

Edemo/tri-n-butylphosphate	10.0	11.0	7890A/5975C	10.3	1.052	7.4
			Intuvo 9000/5977B	11.2	0.328	3.9
	27.0	22.2	7890A/5975C	23.6	0.874	5.8
			EM 640	22.3	0.079	6.5
	8.0	6.1	Intuvo 9000/5977B	5.9	1.232	6.6

Medemo/di-n-amylether	40.0	35.0	7890A/5975C	35.8	1.051	4.8
			EM 640	36.2	0.782	12.3
	10.0	6.5	Intuvo 9000/5977B	6.9	0.429	5.1

**Table 10:** Evaluation of multilaboratory comparison results, showing the determination of several CWAs using the GC/method with the internal standard procedure.

Compound		Tabun	Sarin	Soman	VX agent	Edemo
Known concentration (mg/L)		7.5	9.9	15.0	18.7	23.5
Total number of results		16	24	18	20	20
Accurate/inaccurate results obtained on the system	7890A/5975C	6/0	9/0	8/0	6/0	8/0
	Intuvo 9000/5977B	10/0	8/1	6/0	8/1	6/0
	EM 640	0/0	6/0	4/0	4/1	6/0
Total number of accurate results		16	23	18	18	20
Average determined multilaboratory concentration (mg/L)		7.2	9.7	14.3	19.7	24.8
Relative difference of known and determined concentration (%)		−4.0	−2.0	−4.7	+5.3	+5.5
Relative standard deviation between laboratories (%)		10.0	14.1	9.7	13.3	14.4

## Data Availability

No data were used to support this study.
